# Fusion to *Tetrahymena thermophila* granule lattice protein 1 confers solubility to sexual stage malaria antigens in *Escherichia coli*

**DOI:** 10.1016/j.pep.2018.08.001

**Published:** 2019-01

**Authors:** Alka Agrawal, Yelena Bisharyan, Ashot Papoyan, Janna Bednenko, Joanna Cardarelli, Monica Yao, Theodore Clark, Mehmet Berkmen, Na Ke, Paul Colussi

**Affiliations:** a TetraGenetics Inc, Arlington, MA, USA; b Department of Immunology and Microbiology, Cornell University, Ithaca, NY, USA; c New England Biolabs, Ipswich, MA, USA

**Keywords:** Malaria, *Plasmodium*, Particle-based vaccine, Solubility tag, Bacterial protein expression, *Tetrahymena*, EPA, ExoProtein A, Grl1p, granule lattice protein 1, G-SOME, self-assembling Grl particle, SMFA, standard membrane feeding assay, TBV, transmission-blocking vaccine, VLP, virus-like particle

## Abstract

A transmission-blocking vaccine targeting the sexual stages of *Plasmodium* species could play a key role in eradicating malaria. Multiple studies have identified the *P. falciparum* proteins Pfs25 and Pfs48/45 as prime targets for transmission-blocking vaccines. Although significant advances have been made in recombinant expression of these antigens, they remain difficult to produce at large scale and lack strong immunogenicity as subunit antigens. We linked a self-assembling protein, granule lattice protein 1 (Grl1p), from the ciliated protozoan, *Tetrahymena thermophila*, to regions of the ectodomains of either Pfs25 or Pfs48/45. We found that resulting protein chimera could be produced in *E. coli* as nanoparticles that could be readily purified in soluble form. When produced in the *E. coli* SHuffle strain, fusion to Grl1p dramatically increased solubility of target antigens while at the same time directing the formation of particles with diameters centering on 38 and 25 nm depending on the antigen. In a number of instances, co-expression with chaperone proteins and induction at a lower temperature further increased expression and solubility. Based on Western blotting and ELISA analysis, Pfs25 and Pfs48/45 retained their transmission-blocking epitopes within *E. coli*-derived particles, and the particles themselves elicited strong antibody responses in rabbits when given with an aluminum-based adjuvant. Antibodies against Pfs25-containing nanoparticles blocked parasite transmission in standard membrane-feeding assays. In conclusion, fusion to Grl1p can act as a solubility enhancer for proteins with limited solubility while retaining correct folding, which may be useful for applications such as the production of vaccines and other biologics.

## Highlights

• Malaria antigens were fused with a self-assembling protein from *T. thermophila*.
• Fusion dramatically increased the solubility of Pfs25 and Pfs48/45 in *E. coli*.
• The fusion proteins formed particles and retained correct folding.
• Antibodies raised against the fusion proteins blocked malaria transmission.

## 1 Introduction

Malaria is endemic in many parts of the world, with 216 million reported cases and 445,000 malaria deaths in 91 countries in 2016 according to the World Health Organization [1]. The illness is caused by *Plasmodium* species and is transmitted by female *Anopheles* mosquitoes. *Plasmodium falciparum* accounts for 99% of malaria cases in sub-Saharan Africa, while *Plasmodium vivax* is more common in the Americas, Southeast Asia, and the Eastern Mediterranean. Africa accounted for 91% of all malaria deaths [1]. *Plasmodium* has a complicated life cycle. Sporozoites carried by Anopheles mosquito vectors are injected into a vertebrate host where they invade liver cells and develop into merozoites that then go on to infect red blood cells. Small numbers of blood-stage parasites develop into the sexual stage, or gametocyte, which is taken up by the female mosquito as part of a blood meal. Gametocytes develop into gametes in the midgut of the mosquito, undergo fertilization, and eventually develop into sporozoites, which travel to the salivary glands to initiate a new round of infection [2].

A number of studies have shown that naturally acquired immune responses against the parasite antigens, Pfs48/45 and Pfs230, which are expressed in circulating pre-fertilization gametocytes in humans, can reduce malaria transmission [[3], [4], [5], [6]]. These and a number of additional antigens including Pfs25, which is expressed by zygotes and ookinetes in the mosquito midgut, have been the focus of intense efforts towards the development of transmission-blocking vaccines (TBVs) that would underpin an overall strategy for malaria eradication targeting multiple life cycle stages [7]. However, these proteins have been difficult to produce due to a plethora of disulfide bonds, and in the case of Pfs230, large size. Initial studies showed that antibodies against recombinant forms of Pfs48/45 produced in virus-infected mammalian cells [8], *E. coli* [9], and *Saccharomyces cerevisiae* [10] failed to block transmission. Subsequently, transmission-blocking antibodies were generated against an *E. coli*-produced Pfs48/45 fragment fused to maltose-binding protein and co-expressed with four chaperones [11], as well as codon-harmonized Pfs48/45 produced in *E. coli* [12]. Along with Pfs48/45, Pfs25 has received intense scrutiny as a potential TBV candidate. Pfs25 contains eleven predicted disulfide bonds that have been confirmed and mapped [13] and match the locations in the crystal structure of Pfs25 [14] and the *P. vivax* homolog, Pvs25 [15]. Recombinant Pfs25 capable of inducing transmission-blocking antibodies in animals has been produced in multiple heterologous systems including algae [16], plants [17], cell-free systems [18], insect cells [19], and yeast (initially *Saccharomyces cerevisiae* [20], and then at higher quality in *Pichia pastoris* strains over-expressing protein disulfide isomerase [21]). In *E. coli*, initial efforts at producing Pfs25 as a TrpE fusion resulted in incorrectly folded protein [22], and a more recent attempt failed to produce soluble protein [19]. However, a codon-harmonized gene expressed in *E. coli* and refolded from solubilized inclusion bodies retained reduction-sensitive epitopes and induced transmission-blocking antibodies in immunized mice [23].

Despite these advances, Phase I clinical trials with recombinant protein produced in *Pichia* showed that Pfs25 is a relatively weak antigen [24]. To address this issue, second generation vaccines have attempted to re-engineer the protein to increase its overall immunogenicity. For example, a conjugate vaccine consisting of Pfs25 produced in *P. pastoris* that is chemically conjugated to a non-toxic form of ExoProtein A (EPA) derived from *Pseudomonas aeruginosa* has been shown to significantly increase both the anti-Pfs25 and transmission blocking antibody response in rodents [25]. Interestingly the Pfs25-EPA conjugate assumes a particulate form composed of nanoparticles with a diameter of 10–50 nm as determined by atomic force microscopy and dynamic light scattering [25]. Similarly, *P. pastoris*-produced Pfs25 chemically conjugated to the outer-membrane protein complex of *Neisseria meningitidis* serogroup B was more immunogenic than Pfs25 alone [26]. In another approach, virus-like particles consisting of Pfs25 fused to the Alfalfa mosaic virus coat protein and produced in tobacco induced potent transmission-blocking immune responses in mice when adjuvanted with Alhydrogel [27]. Pfs25 fused to the nanoparticle-forming protein IMX313 and produced from viral vectors or in *P. pastoris* showed better antibody responses in mice and better transmission-blocking activity compared with monomeric Pfs25 [28].

From a manufacturing standpoint, *E. coli* remains a preferred platform for the production of subunit vaccine antigens owing to its robust expression characteristics, scalability, and long history with regulatory approval [23]. However, one of its principal drawbacks, particularly for the production of highly disulfide-bonded parasite antigens, is its propensity for protein misfolding. In the case of TBV candidates, this leads to the formation of insoluble inclusion bodies, which must be denatured and refolded to reconstitute the conformation-dependent epitopes that are generally required for protective immunity [23]. Protein refolding can be a cumbersome, inefficient process that directly impacts manufacturing costs, an important factor governing vaccine implementation in the developing world [29]. Potentially, this issue can be overcome by increasing the solubility of expressed proteins, their relative immunogenicity, or both.

Recently, our laboratory has explored the use of self-assembling proteins from the ciliated protozoan, *Tetrahymena thermophila*, for the production of particle-based vaccines. This approach utilizes so-called granule lattice proteins (Grls) from *Tetrahymena* as fusion tags. Grls are acidic calcium-binding proteins with predicted coiled-coil protein interaction domains that form crystalline arrays within the dense core secretory mucocysts of *T. thermophila* [30], and are capable of self-assembly to form nanoparticles (termed G-SOMEs) *in vitro* under various buffer conditions (Y. Bisharyan and T. Clark, unpublished data). With the idea that antigens linked to these proteins could assemble into higher order structures in heterologous systems, we engineered fusion constructs juxtaposing regions of the ectodomains of Pfs25 and Pfs48/45 with Grl1p, the major granule lattice protein of *Tetrahymena*, and expressed these in *E. coli.* In the current study, we show that the resulting chimera self-assembled into particles with peak diameters centering on 38 and 25 nm, respectively. Additionally, and perhaps more importantly, Grl1p increased the solubility of the recombinant antigens by more than 100-fold allowing high-yield purification of recombinant fusion constructs with no need for protein refolding. Malaria antigens retained their conformation-dependent transmission-blocking epitopes in the *E. coli*-derived particles, and Pfs25-containing G-SOMEs generated high-titer transmission blocking antibody production in test animals.

## 2 Materials and methods

### 2.1 Materials

Mouse monoclonal antibody 4B7 against Pfs25 [20] was obtained through the Malaria Research and Reference Reagent Resource Center at the NIH. Monoclonal antibody 3E12 against Pfs48/45 [31] was obtained from Dr. Nicholas MacDonald (Malaria Vaccine Branch, NIH). Monoclonal antibodies 1G2 and 4F7 against Pfs25 were obtained from Dr. David Narum and Dr. Patrick Duffy (Laboratory of Malaria Immunology and Vaccinology, NIH) [25]. Mouse monoclonal antibody 32F81 against Pfs25 [32], rat monoclonal antibodies 85RF45.1 and 85RF45.2b against Pfs48/45 [33], and purified *P. falciparum* gametes and gametocytes were obtained from TropIQ Health Sciences (Nijmegen, the Netherlands).

### 2.2 Expression construct design

Pfs25, Pfs48/45^10C^, and Grl1p genes were synthesized by a commercial vendor (Genscript). Coding sequences for the Pfs25 (amino acids 22–193) and Pfs48/45 10C fragments (amino acids 159–426 [11]) lacking native N- and C-terminal signal peptide domains, were codon-harmonized for expression in *E. coli* as described previously [12,23]. Similarly the *Tetrahymena thermophila* Grl1p gene minus the coding region for the N-terminal signal peptide (amino acids 19–402; accession number AAB19104.1) was codon-optimized for expression in *E. coli*. Pfs25 and Pfs48/45 coding sequences were modified with a 5′ HA tag (YPYDVPDYA) and a 3′ 10X histidine tag. For fusions of Pfs25 and Pfs48/45^10C^ with Grl1p, the coding sequence of Grl1p was placed at the 3′ end with a BamHI site linking the two genes. Fusion genes were also modified with 5′ HA and 3’ 10X His tags. All genes were cloned into vector pET21a (EMD Millipore, Billerica, MA).

The chaperone genes consisted of the human endoplasmic reticulum chaperone BiP [34] (amino acids 19–654, accession number NP_005338.1), the *E. coli* periplasmic serine endoprotease DegP1(S210A) [35] (amino acids 27–474, accession number NP_414703), the *E. coli* peptidyl-prolyl cis-trans isomerase surA [36] (amino acids 21–428, accession number NP_414595), the *E. coli* peptidyl-prolyl cis-trans isomerase fkpA [37] (amino acids 26–270, accession number NP_417806), the *E. coli* peptidyl-prolyl cis-trans isomerase trigger factor fused to fkpA (Tig-fkpA) [37,38] (Tig amino acids 1–143, accession number NP_417806, fkpA as described), the *S. cerevisiae* protein disulfide-isomerase MPD1 [39] (amino acids 22–318, accession number NP_014931.3), the *S. cerevisiae* protein disulfide-isomerase MPD2 [39] (amino acids 25–277, accession number NP_014553.1), and the *E. coli* protein disulfide isomerase DsbC [40] (amino acids 21–236, accession number NP_417369.1). Chaperone genes were cloned into pBAD34 with the exception of Tig-fkpA, which was cloned into pBAD33 [41].

### 2.3 Expression and protein purification

The *E. coli* SHuffle strain (New England Biolabs, catalog #C3029), which has been engineered for improved production of disulfide-bonded proteins in the cytoplasm [42], was transformed with individual plasmids and grown at 30 °C unless otherwise stated. Overnight cultures in Luria-Bertani broth containing ampicillin were diluted 1:100 with the same medium and shaken at 250 rpm. When cultures reached an OD_600_ of 0.5, protein expression was induced with 0.1 mM IPTG for either 4 h at 30 °C or overnight at 16 °C. Bacterial cells were collected by centrifugation and pellets were stored at −80 °C until purification (for proteins induced at 30 °C, except Pfs25 induced at 16 °C) or solubility analysis (for proteins induced at 30 °C and 16 °C).

Bacterial pellets were resuspended in 20 mM Tris, 300 mM NaCl, 5% glycerol, 1% Tween-80, and 30 mM imidazole (pH 8.0) containing protease inhibitors (Roche cOmplete EDTA-free). Bacteria were disrupted in a Microfluidics M-110P microfluidizer for 4 passes at 20,000 pounds per square inch and centrifuged at 21,000 × g for 1 h at 4 °C to remove insoluble material. The supernatant was filtered through a 0.2 μm filter and his-tagged proteins purified at 4 °C by fast protein liquid chromatography on Nickel Sepharose 6 Fast Flow (GE Healthcare) with step gradients up to 1 M imidazole. The protein peak eluting at 250 mM imidazole was pooled and dialyzed extensively against 20 mM Tris, 100 mM NaCl, 5% glycerol (pH 8.0). The final protein was filtered through a 0.2 μm filter, quick frozen in liquid nitrogen, and stored at −80 °C. Protein identities were confirmed by micro-capillary LC/MS/MS (Taplin Mass Spectrometry Facility, Harvard Medical School).

For studies with chaperone proteins, plasmids containing the chaperones BiP, DegP1(S210A), surA, fkpA, Tig-fkpA, MPD1, MPD2, or DsbC were each co-transformed with pET21a-Pfs25Grl1 into the SHuffle strain of *E. coli* (New England Biolabs). Clones containing both plasmids were obtained by selection on 100 μg/ml ampicillin and 34 μg/ml chloramphenicol. Pfs25:Grl1p expression was induced as described above, except that l-arabinose was added to 0.2% when diluting the overnight culture 1:100 to induce expression of the helper plasmid. Pfs25:Grl1p expression was induced for 4 h at 30 °C or 18 h at 16 °C after the addition of IPTG. Total protein production was estimated based on in-gel densitometry compared with bovine serum albumin analyzed with GeneTools (Syngene USA, Frederick, MD). The soluble fraction was obtained by microfluidization and centrifugation as described above, and percent solubility was assessed by Western blot of equal volumes of total and soluble material using an antibody against the C-terminal histidine tag (Invitrogen).

### 2.4 Dynamic light scattering (DLS)

DLS was performed using a Malvern ZS90 Zetasizer (Westborough, Massachusetts). Purified protein samples (0.12–0.2 mg/ml in 20 mM Tris, 100 mM NaCl, 5% glycerol, pH 8.0) were centrifuged at 17,000 × g for 10 min prior to each analysis. Seventy microliter samples were measured six successive times at 4 °C. For particle stability assessments measurements were carried out at 40 °C.

### 2.5 SDS-PAGE and western blot analysis

Purified proteins were mixed with SDS-PAGE loading buffer (with or without DTT), boiled for 5 min, and separated on anykD™ TGX™ gels (BioRad). After transfer to nitrocellulose, blots were blocked with 5% nonfat dry milk (BioRad) in PBS containing 0.05% Tween-20 (PBST) and incubated with antibodies overnight at 4 °C. After incubation with horseradish peroxidase (HRP)-conjugated secondary antibody, blots were developed using SuperSignal West Pico Chemiluminescent Substrate (Thermo Scientific). Blotting of *P. falciparum* gametes and gametocyte extracts was identical except that blots were blocked and antibodies were diluted in PBST containing 2% BSA.

### 2.6 ELISAs

To test the reactivity of Pfs25 samples to antibodies against linear and conformational epitopes on native Pfs25, ELISAs were carried out essentially as described [25]. Recombinantly-expressed Pfs25 (33.3 ng) and Pfs25:Grl1p (100 ng) were incubated overnight on Nunc Maxisorp plates so that wells contained equimolar amounts of the Pfs25 moiety. Plates were washed with PBST and blocked with 2% BSA/PBST for 1 h at RT. Proteins were incubated with either 32F81, 1G2, 4F7, or anti-FLAG (Thermo Fisher) antibodies in a 5-fold dilution series (0.4 μg/ml to 0.000128 μg/ml) in 2% BSA/PBST for 2 h at RT. After washing with PBST, goat anti-mouse HRP (BioRad) diluted 1:4000 in 2% BSA/PBST was added for 1 h at RT. After washing with PBST, plates were developed with 1-Step Ultra TMB reagent (Thermo Scientific).

### 2.7 Transmission electron microscopy

G-SOMEs were negatively stained with 1% sodium silicotungstate on continuous carbon-film grids, and electron micrographs of single particles were recorded using an Ultrascan4000 charge coupled device camera (Gatan, Pleasanton, CA) at 93,000x nominal magnification. Particles were then automatically selected by a computational screening function provided by the proprietary data processing software package PARTICLE (Angstrom BioImaging, Cambridge, MA, http://www.image-analysis.net/EM/), from which statistics on the particle size and other morphological descriptors were generated.

### 2.8 Immunizations

Rabbit immunizations were performed at the Harvard University monoclonal antibody core facility using an IACUC approved protocol. Twenty-four New Zealand White rabbits were separated into four groups of six rabbits each, one group for each recombinant immunogen (Grl1p; Pfs25; Pfs25:Grl1p; Pfs48/45^10C^:Grl1p). All 24 rabbits were pre-bled (5 ml) on Day 0. Two rabbits from each group were immunized with either no adjuvant, Imject Alum (Thermo Scientific, Rockford, IL), or Montanide ISA 720 (SEPPIC, Fairfield, NJ) adjuvants mixed with equal volumes of immunogen (80 μg/rabbit) administered subcutaneously and intramuscularly at five different sites. Additional boosts were given subcutaneously and intramuscularly on days 28 and 56, with bleeds taken from the central ear artery on days 28, 42 and a terminal bleed on day 70. The blood was clotted overnight, and then centrifuged to separate the serum from the clot.

### 2.9 Anti-Grl1p, Pfs25, and Pfs48/45^10C^ end-point titer ELISA

For Pfs25 and Grl1p end-point titer determination, plates (96-well Nunc Maxisorp U-bottom) were coated in triplicate with 50 μl of purified bacterial protein at 2 μg/ml in PBS overnight at 4 °C. All subsequent steps were carried out at room temperature and all washes were performed with PBST. Plates were washed and blocked with PBST containing 2% bovine serum albumin for 1 h. Starting dilutions of 1/5,000, 1/30,000 and 1/200,000 for sera samples collected on days 28, 42 and 70, respectively, were added to microtiter wells. Two-fold serial dilutions of sera in blocking buffer were incubated with protein for 2 h, washed, and incubated with goat anti-rabbit HRP (Bio-Rad) in blocking buffer for 1 h. After washing, plates were developed with 1-Step Ultra TMB reagent (Thermo Scientific) for 15–30 min. Reactions were stopped with an equal volume of 2 M sulfuric acid and absorbances were read at 450 nm. Endpoint titers were defined as the highest dilution of the immune serum that gave an absorbance above the mean plus three standard deviations of the same dilution of the pre-immune serum.

Due to a lack of availability of soluble Pfs48/45^10C^, anti-Pfs48/45^10C^ end-point titers were determined in ELISAs following depletion of anti-Grl1p antibodies from day 42 serum samples. Serum samples diluted 1:5000 in 2% BSA/PBST were incubated with 40 μg recombinant Grl1p attached to Ni Sepharose beads overnight at 4 °C. Beads were removed by centrifugation and serum supernatants tested on ELISA plates coated with either recombinant Grl1p (to confirm anti-Grl1p depletion) or Pfs48/45^10C^:Grl1p (to determine anti-Pfs48/45^10C^ titer).

### 2.10 Standard membrane feeding assay (SMFA)

Total rabbit IgG was purified from sera by Protein G affinity chromatography and adjusted to a concentration of 20 mg/ml in phosphate-buffered saline. A 200 μl mixture containing mature *in vitro* cultured gametocytes of *P. falciparum* (NF54 isolate) and normal human serum (50% hematocrit) was mixed with 60 μl of purified rabbit IgG and fed to 3–6 day old female *Anopheles stephensi* Nijmegen mosquitoes through a membrane-feeding apparatus. The assay was performed without human complement and the final IgG concentration in a feeder was 7.5 mg/ml. After 8 days, 20 mosquitoes were dissected and the number of mature oocysts in the midgut was determined. Only midguts from mosquitoes with any eggs at the time of dissection were analyzed to exclude unfed mosquitoes. 4B7 antibody at 94 μg/ml was used as a positive control to show >80% transmission blocking. The human serum and red blood cells used for the cultures were purchased from Key Biologics (Memphis, TN) and Interstate Blood Bank (Memphis, TN). The 95% confidence intervals (CI) of percent inhibition of mean oocyst intensity and p-value were calculated based on a zero-inflated negative binomial model as described previously [43].

## 3 Results

### 3.1 Expression and purification of Pfs25:Grl1p and Pfs48/45^10C^:Grl1p fusions in *E. coli*

We reasoned that the self-assembly properties of *Tetrahymena* Grl1p would allow production of particle-based transmission blocking vaccines for malaria in a well-validated, robust, and inexpensive host expression platform. Chimeric genes in which the coding sequence of the Grl1p proprotein was placed downstream of the coding regions of either Pfs25, or the 10C fragment of Pfs48/45 (herein referred to as Pfs48/45^10C^) were prepared. The 10C fragment was chosen based on a previous report of its increased stability in *E. coli* relative to full length Pfs48/45 protein [11]. Gene fragments were codon-harmonized according to Kumar et al. [12,23], and fusion constructs were expressed in the SHuffle strain of *E. coli* [42]) engineered for improved production of disulfide-bonded proteins in the cytoplasm.

Consistent with previous findings [12,19,23] both Pfs25 and Pfs48/45^10C^ were largely insoluble when expressed in *E. coli* at 30 °C, although very small amounts of Pfs25 remained soluble after induction at 16 °C. By contrast, significant amounts of Pfs25:Grl1p and Pfs48/45^10C^:Grl1p fusion proteins produced at 30 °C were detected in filtered extracts indicating that Grl1p conferred improved solubility to each of the antigens. In the case of the Pfs25:Grl1p fusion, approximately 180-fold more soluble protein was recovered compared with Pfs25 alone when both were induced at 16 °C (13.5 mg versus 0.075 mg per liter, respectively, see Table 4). Although similar comparisons of fold increase were not possible for Pfs48/45^10C^ and Pfs48/45^10C^:Grl1p due to the lack of recovery of any quantifiable amounts of soluble Pfs48/45^10C^, similar amounts of soluble Pfs48/45^10C^:Grl1p were easily obtained per liter of non-optimized culture (A. Agrawal, unpublished results).

**Table 1:** Average DLS volume distribution measurements of G-SOME particles.

G-SOME protein	Peak diameter by volume	Percent by volume
Pfs25	10	100
Pfs25:Grl1p	38	40
	106	60
Pfs48/45^10C^:Grl1p	25	50
	70	50
Grl1p	13	100

**Table 2:** **Pfs25, Pfs25:Grl1p, Pfs48/45**^**10C**^**Grl1p, and Grl1p end-point titers**. Titers for each of the two rabbits are given (in thousands).

Immunization Antigen	Adjuvant	**ELISA Antigen**^a^	Day 28	Day 42	Day 70
Pfs25	None	Pfs25	16; 32	480; 3840	<200; 1600
	Alum	Pfs25	64; 128	3840; 3840	12,800; 25,600
	Montanide	Pfs25	1040; 1040	3840; 3840	12,800; 25,600
Pfs25:Grl1p	None	Pfs25	0.1; 0.1	60; 120	<200; <200
	Alum	Pfs25	240; 30	3840; 3840	800; 25,600
	Montanide	Pfs25	240; 240	3840; 3840	3200; 25,600
Pfs48/45^10C^:Grl1p	None	Pfs48/45^10C^:Grl1p	ND^b^	<5, 20	ND^b^
	Alum	Pfs48/45^10C^:Grl1p	ND^b^	10, 160	ND^b^
	Montanide	Pfs48/45^10C^:Grl1p	ND^b^	10, 640	ND^b^
Grl1p	None	Grl1p	0.2; 25	120; 960	<200; <200
	Alum	Grl1p	1250; 1250	3840; 3840	12,800; 6400
	Montanide	Grl1p	2500; 2500	3840; 3840	6400; 25,600

a For Pfs48/45^10C^:Grl1p, ELISAs were done for day 42 bleeds only after preadsorbing sera to Grl1p to remove antibodies against Grl1p.

b ND, not done.

**Table 3:** **Standard membrane feeding assay (SMFA)**. Sera from the higher titer rabbit at day 70 were analyzed. Titers at day 42 were used to determine higher titer rabbit for Pfs48/45^10C^:Grl1p.

Immunization Antigen	Adjuvant	Percent Inhibition	95% CI^a^ Low	95% CI^a^ High	p value	n^b^
Pfs25	Alum	98.0	97.4	99.7	0.001	2
Pfs25:Grl1p	Alum	82.9	61.9	92.3	0.001	2
Pfs25:Grl1p	Montanide	67.8	30.0	85.6	0.003	2
Pfs48/45^10C^:Grl1p	Montanide	39.4	−13.5	68.6	0.118	3
Grl1p	Montanide	16.8	−56.9	55.0	0.602	3

a CI, confidence interval.

b n, number of experiments.

**Table 4:** Effect of chaperones and temperature on Pfs25 and Pfs25:Grl1p expression and solubility.

Protein	Chaperone	Induction Temperature (°C)	Induction Time (hours)	Total Protein (mg/L)	Percent Soluble	Soluble Protein (mg/L)
Pfs25	None	30	ND^a^	ND^a^	ND^a^	ND^a^
		16	18	30.0	0.25	0.075
Pfs25:Grl1p	None	30	4	40.7	1.4	0.6
		16	18	72.8	18.6	13.5
Pfs25:Grl1p	BiP	30	4	48.7	<1	0.05
		16	18	92.8	27.2	25.2
Pfs25:Grl1p	DegP1 (S210A)	30	4	42.1	<1	0.04
		16	18	86.3	18.1	15.6
Pfs25:Grl1p	surA	30	4	69.4	10.2	7.1
		16	18	113.1	31.2	35.3
Pfs25:Grl1p	fkpA	30	4	60.4	3.1	1.9
		16	18	55.1	56.0	30.9
Pfs25:Grl1p	Tig-fkpA	30	4	81.7	5.0	4.1
		16	18	65.8	49.5	32.6
Pfs25:Grl1p	MPD1	30	4	67.9	2.8	1.9
		16	18	110.3	45.4	50.1
Pfs25:Grl1p	MDP2	30	4	41.3	5.0	2.1
		16	18	38.2	41.1	15.7
Pfs25:Grl1p	DsbC	30	4	34.5	2.2	0.8
		16	18	42.5	66.9	28.4

a ND, not done.

**Fig. 1:**
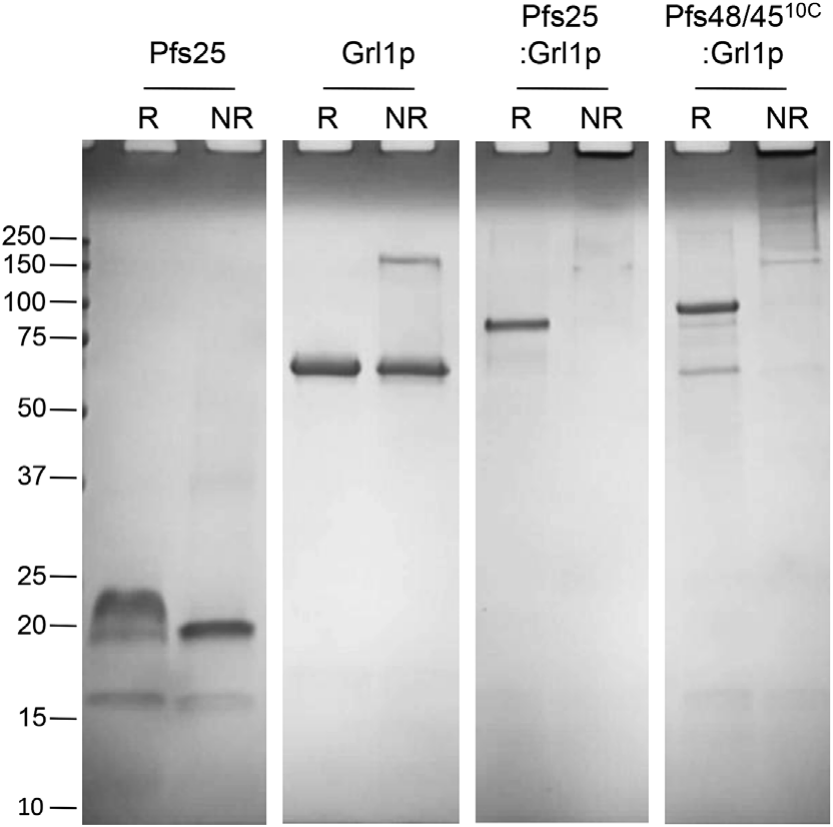
**SDS-PAGE analysis of purified proteins**. Pfs25, Grl1p, Pfs25:Grl1p, and Pfs48/4510C:Grl1p were expressed in *E. coli* and purified using nickel sepharose. Proteins (2 mg per lane) were separated under reducing (R) or nonreducing (NR) conditions and stained with Coomassie. Relative position of molecular weight markers (kDa) are shown on left.

**Fig. 2:**
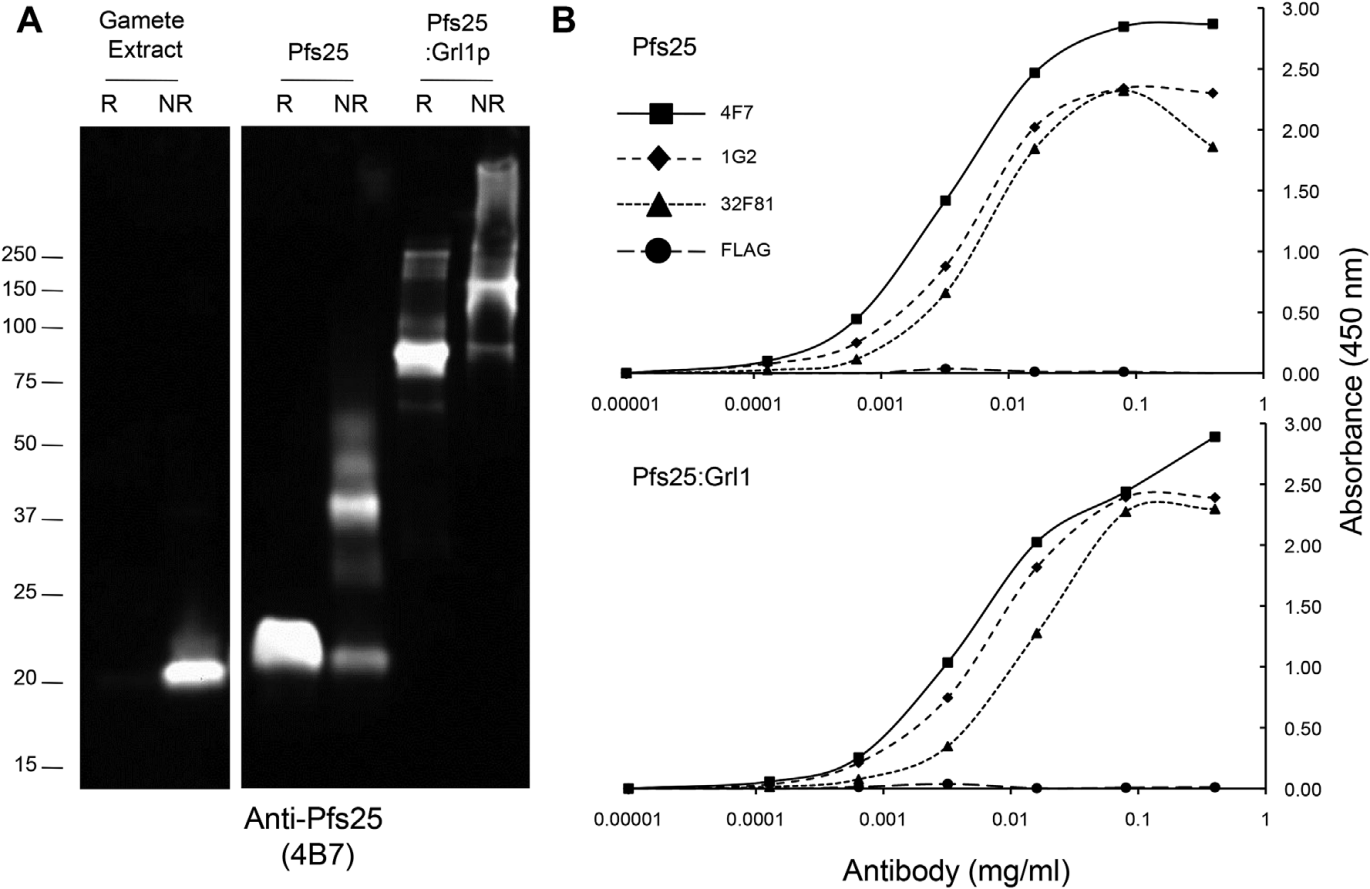
**Characterization of Pfs25 and Pfs25:Grl1p epitopes**. A. Anti-Pfs25 Western Blot. Purified P. falciparum gametes (500,000 per lane), and 1 μg Pfs25 and Pfs25:Grl1p were resolved by SDS-PAGE under reducing (R) or non-reducing (NR) conditions and analyzed by Western blot with the transmission-blocking mAb 4B7. B. ELISA titration of anti-Pfs25 transmission-blocking antibodies. Pfs25 (Top Panel) and Pfs25:Grl1p (Bottom Panel) were adsorbed to microtiter wells in equimolar amounts with respect to Pfs25. Serial 5-fold dilutions of antibodies that recognize either a linear (32F81) or conformational (1G2, 4F7) Pfs25 antibodies were applied to wells. A non-related antibody (FLAG) was included as a negative control in each analysis.

**Fig. 3:**
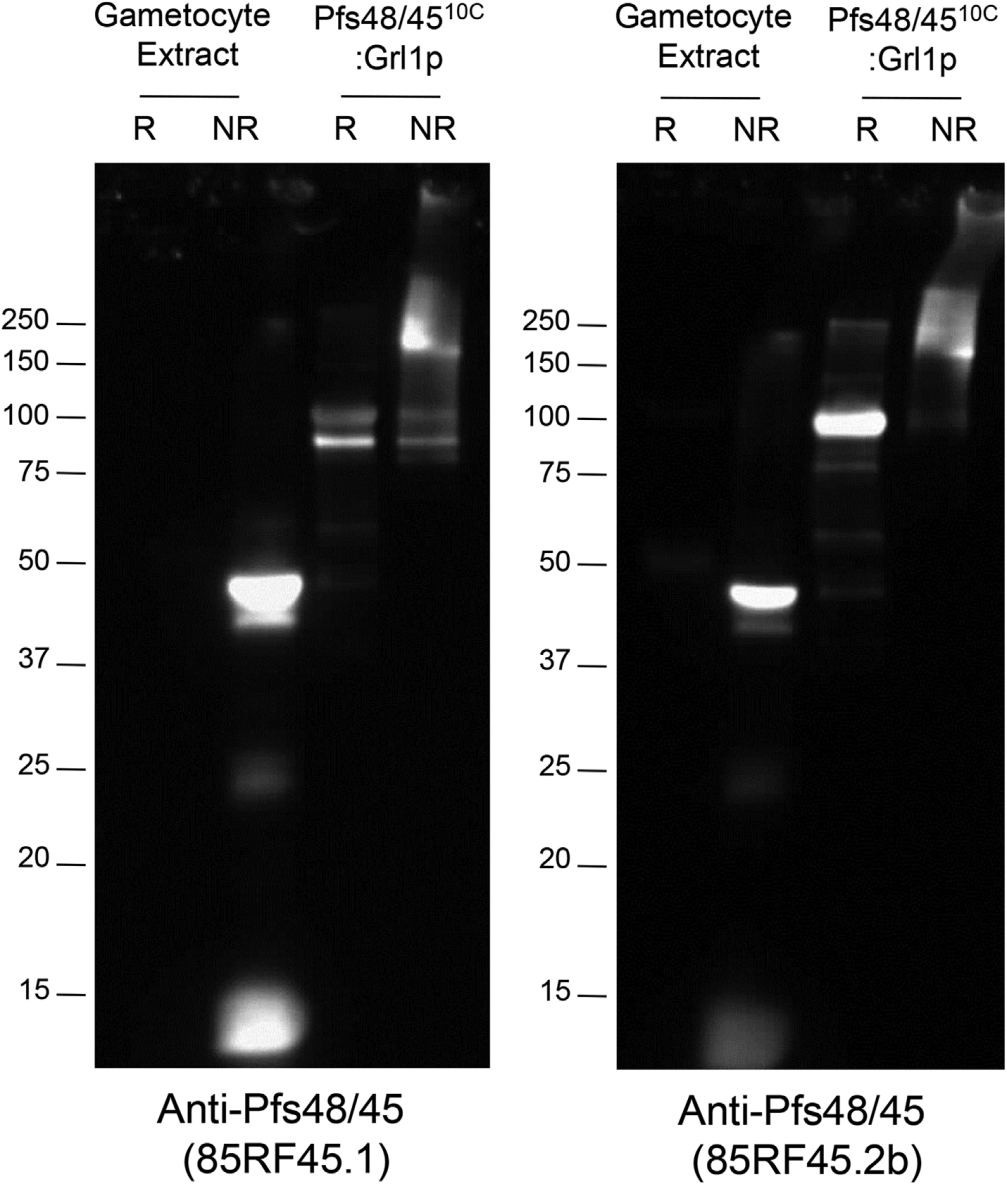
**Characterization of Pfs48/4510C:Grl1 epitopes**. Purified P. falciparum gametocytes (330,000 per lane) and Pfs48/45^10C^:Grl1p (1 μg per lane) were resolved by SDS-PAGE under reducing (R) or non-reducing (NR) conditions and analyzed by Western blot with the transmission-blocking mAb 85RF45.1 (Left Panel) and the conformational but non-transmission blocking mAb 85RF45.2b (Right Panel). The position of molecular weight markers are indicated.

Histidine-tagged Pfs25:Grl1p, Pfs48/45^10C^:Grl1p, and Grl1p alone were expressed at 30 °C, purified by nickel chromatography, and analyzed by SDS-PAGE under reducing and non-reducing conditions (Fig. 1). As Pfs25 was insoluble when expressed at 30 °C, the protein was purified from bacteria induced at 16 °C. Under reducing conditions, Pfs25:Grl1p and Pfs48/45^10C^:Grl1p fusion proteins resolved with apparent molecular masses of 80 kDa and 100 kDa, respectively, somewhat higher than their predicted masses due to the acidic nature of the Grl1p fusion partner [30]. Under non-reducing conditions, however, both fusion constructs resolved as low-mobility smears with a portion of the proteins remaining in the well, consistent with the formation of disulfide-dependent higher-order structures. Recombinant Pfs25 alone resolved with an apparent mass of 20 kDa under non-reducing conditions and ∼25 kDa under reducing conditions. The lower molecular weight bands are likely contaminants, since they show little to no reactivity with antigen-specific antibodies (see Fig. 2, Fig. 3). The recombinant Grl1p fusion tag resolved at an apparent molecular mass of ∼68 kDa under both reducing and non-reducing conditions.

### 3.2 Conformational epitopes are present in Pfs25:Grl1p and Pfs48/45^10C^:Grl1p

As a measure of whether the recombinant fusion proteins were correctly folded, we determined whether transmission-blocking epitopes were formed and maintained. Fig. 2 shows an analysis of Pfs25 proteins by Western blot and ELISA with a variety of transmission blocking monoclonal antibodies (mAbs). We used mAbs that recognize either conformation-dependent epitopes (1G2, 4F7) [25], or a linear, reduction-sensitive epitope (4B7) [20,44]. As shown in Fig. 2A, antibody 4B7 reacted differently with Pfs25 derived from different sources. As expected, native Pfs25 from *P. falciparum* gamete extracts was only recognized under non-reducing conditions, indicating correctly folded protein. In contrast, reactivity of 4B7 against *E. coli*-expressed Pfs25 and Pfs25:Grl1p was largely dependent on reducing conditions. 4B7 reacted more strongly under non-reducing conditions against the Pfs25:Grl1p fusion protein (∼40%) than against Pfs25 alone (∼25%), suggesting that the chimeric gene product retains more of the native fold associated with the 4B7 epitope. Finally, 4B7 failed to react with either Grl1p or Pfs48/45^10C^:Grl1p as expected (data not shown).

To further characterize recombinant Pfs25, antibody titrations were carried out by ELISA with both linear (32F81) and conformation-dependent (1G2, 4F7) mAbs. Titration curves showed that *E. coli* derived Pfs25 and Pfs25:Grl1p were detected with virtually the same efficiency regardless of the antibody (Fig. 2B) indicating soluble Pfs25 expressed in *E. coli* maintains conformational epitopes, and more importantly, that fusion to Grl1p does not interfere with the formation of these epitopes. These results are similar to those observed for the Pfs25 and the Pfs25:EPA conjugate expressed in *Pichia pastoris,* where the Pfs25 moiety was recognized by mAbs 4B7, 1G2, and 4F7 at roughly equal titrations [25].

To probe the epitope structure of the Pfs48/45 fusion protein, mAb 85RF45.1, which recognizes epitope 1 on the native parasite protein and blocks transmission, and mAb 85RF45.2b, which recognizes epitope 2b and does not block transmission [33], were examined by Western blot analysis. As shown in Fig. 3, both antibodies detected native Pfs48/45 in gametocyte extracts only under non-reducing conditions, as expected for correctly folded protein. The same antibodies recognized *E. coli* derived Pfs48/45^10C^:Grl1p under both reducing and non-reducing conditions, with approximately 60% and 30% of total reactivity detected under non-reducing conditions for antibodies 85RF45.1 and 85RF45.2b, respectively. Similar results were observed with the epitope 1 reactive mAb, 3E12 [31] (data not shown), indicating that at least a portion of Pfs48/45 fusion protein maintained reduction-sensitive epitopes following expression in *E. coli*.

### 3.3 Pfs25:Grl1p and Pfs48/45^10C^:Grl1p form G-SOME particles

Recombinant Grl1p fusion proteins expressed in *Tetrahymena* assemble into discrete G-SOME particles (Y. Bisharyan and T. Clark, unpublished data). To investigate whether similar assemblies were being produced in *E. coli* we analyzed purified recombinant Grl1p, Pfs25, Pfs25:Grl1p, and Pfs48/45^10C^:Grl1p by dynamic light scattering (Table 1). Average volume size distributions from multiple (>5) consecutive measurements showed that *E. coli* derived Grl1p and Pfs25 formed 13 and 10 nm particles, respectively. However, Pfs25:Grlp and Pfs48/45^10C^:Grl1p each showed two volume size distribution peaks of 38 nm and 106 nm and 25 nm and 70 nm, respectively.

**Fig. 4:**
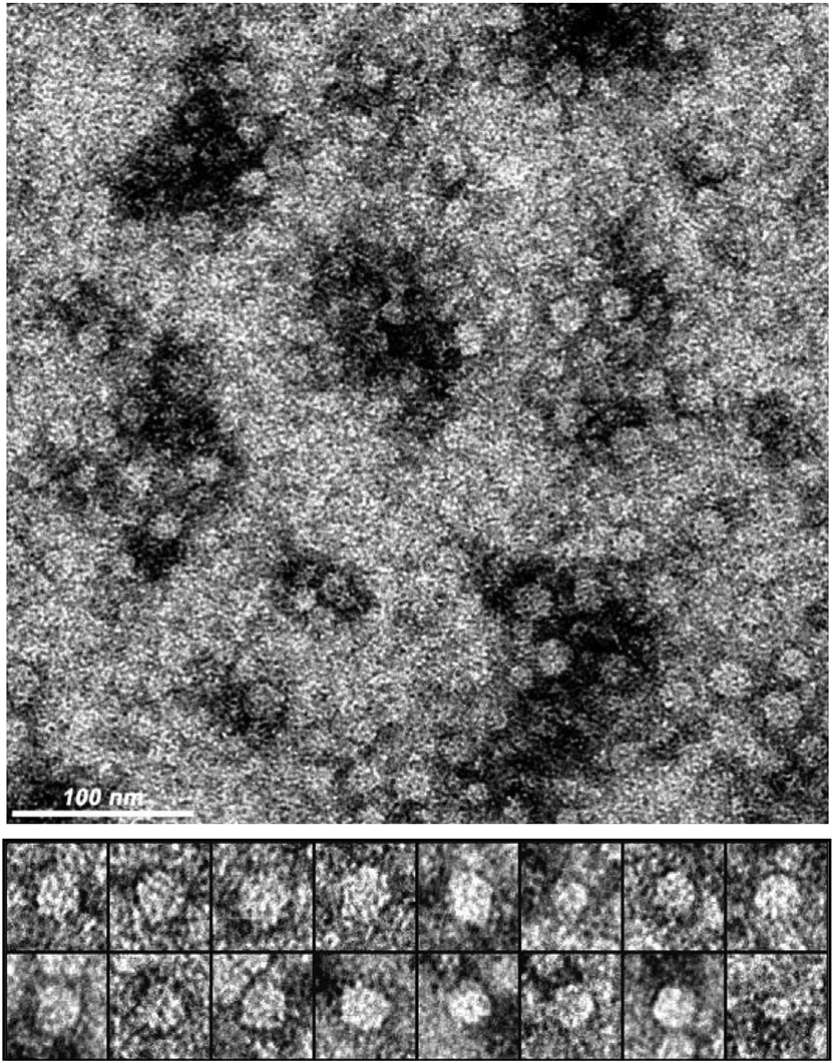
**Transmission electron microscopy of Pfs25:Grl1p**. Purified Pfs25:Grl1p was negatively stained with 1% sodium silicotungstate and observed at 93,000x magnification. The top panel shows a field of view with 100 nm size bar at the bottom left. The bottom panel shows a close up image of 16 individual particles.

To confirm the DLS results and determine the morphology of G-SOME particles, we analyzed Pfs25:Grl1p G-SOMEs by negative stain electron microscopy (Fig. 4). The results indicate that the protein forms structured globular particles approximately 20 nm in diameter (range, 12–28 nm), with more than 90% forming ordered assemblies, and less than 5% nonspecific aggregates. None of the larger particles detected by DLS were observed by EM perhaps reflecting selective adherence of smaller particles to the grids and/or the significantly higher ratio of smaller to larger particles in the sample.

### 3.4 Immunization of rabbits elicits high titer antibody production

To determine the immunogenicity of soluble and particulate forms of Pfs25 and Pfs48/45^10C^ antigens, rabbits were immunized 3 times with 80 μg doses of protein either with or without adjuvant over a 70-day period using protocols described previously for transmission blocking vaccine candidates [45]. In the case of soluble and particulate Pfs25, very high antibody titers were achieved in all animals receiving the adjuvanted samples with levels peaking at 42 days in some animals (Table 2). Regardless of the antigen, antibody titers were always higher in animals that received the adjuvanted samples. Somewhat surprisingly, no differences in the peak titers were observed in animals injected with soluble Pfs25 versus Pfs25 in G-SOMEs, either with or without adjuvant, suggesting that the particulate nature of the antigen did not significantly boost antigenicity. In the case of Pfs48/45^10C^, the absence of a soluble form of the antigen from *E. coli* obviated such comparisons and also complicated ELISA-based assays to determine specific antibody titers in animals injected with the particulate Pfs48/45^10C^:Grl1p antigen. Initial attempts at ELISA used gametocyte extracts as the source of the antigen on plates, however, high backgrounds with pre-immune sera obscured the results in all but one case (day 42 serum from one animal; data not shown). Therefore, day 42 sera were pre-adsorbed with recombinant Grl1p to remove anti-Grl1p specific antibodies and then examined using Pfs48/45^10C^:Grl1p as the test antigen. Serum from one of the animals in the Montanide group had a significantly higher anti-Pfs48/45 titer compared to the other samples and was chosen for further analysis (see below). These results also appeared to indicate that unlike the response to Pfs25:Grl1p, a majority of the immune response to Pfs48/45^10C^:Grl1p targeted the Grl1p moiety over the Pfs48/45^10C^ antigen. In general, sera from immunizations with either fusion protein had titers towards recombinant Grl1p that were similar to titers in sera from immunizations with Grl1p alone (data not shown).

### 3.5 Sera from immunized rabbits recognize the corresponding native proteins

**Fig. 5:**
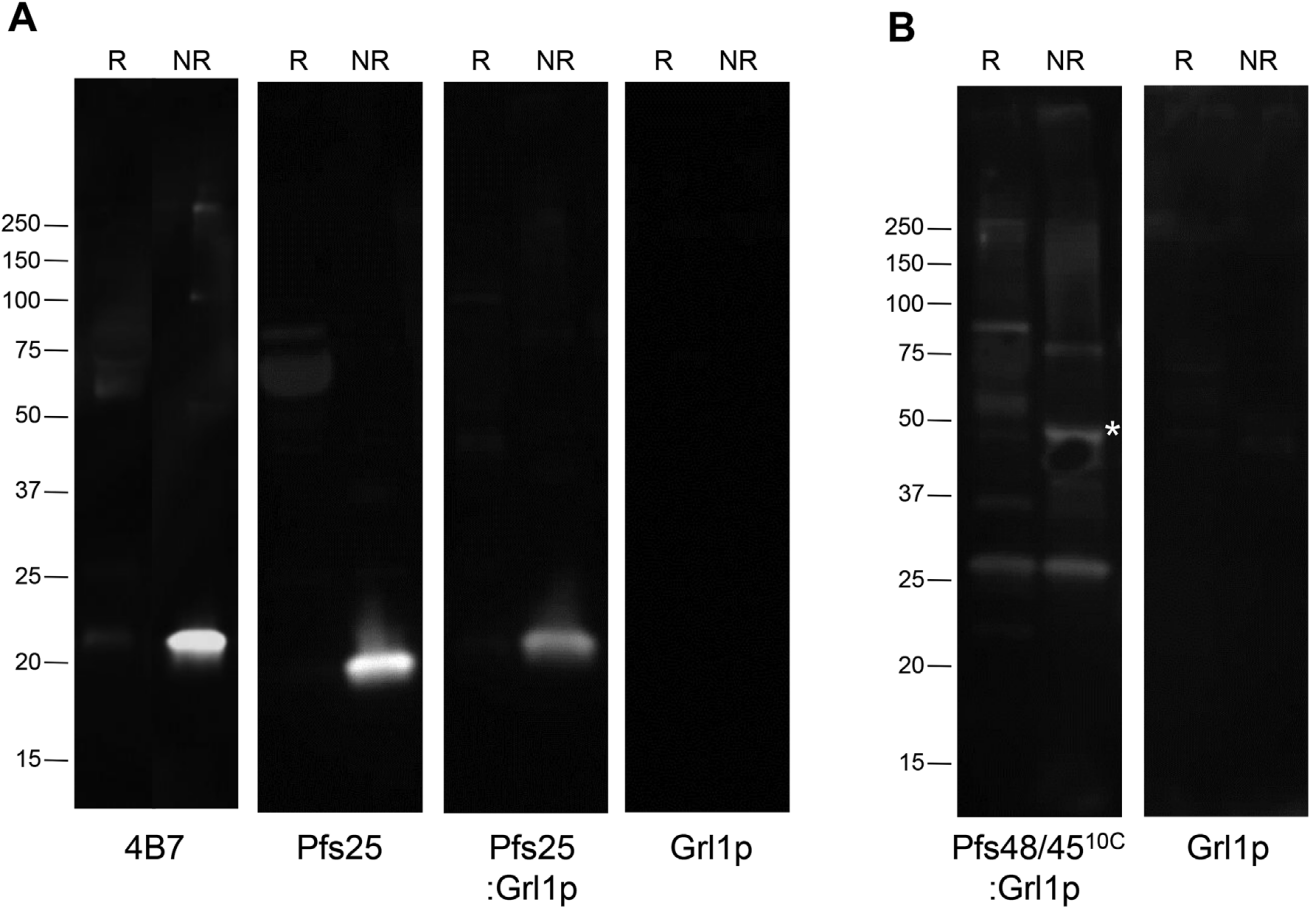
**Reactivity of 4B7 antibody and sera against native Pfs25 and Pfs48/45. P**. *falciparum* gametes and gametocytes were resolved by SDS-PAGE under reducing (R) and non-reducing (NR) conditions and analyzed by Western blot. A. Western blot of gamete extract (250,000 per lane) with 4B7 antibody, and select day 70 sera raised against Alum adjuvanted Pfs25, Alum adjuvanted Pfs25:Grl1p, and Alum adjuvanted Grl1p. B. Western blot of gametocyte extract (250,000 per lane) with select day 70 sera raised against Montanide adjuvanted Pfs48/4510C:Grl1p and Grl1p. The position of molecular weight markers are as indicated. Assumed Pfs48/45 band is highlighted with an asterix.

Western blotting analysis was carried out to determine whether antibodies directed against the recombinant antigens recognized the native Pfs25 and Pfs48/45 antigens from gamete or gametocyte extracts, respectively. As shown in Fig. 5, sera from rabbits immunized with either soluble or particulate forms of Pfs25 detected a single protein of the expected mass of Pfs25 in gamete extracts, and only under non-reducing conditions (Fig. 5A) essentially duplicating the results with the transmission blocking antibody, 4B7 (Fig. 5A, left most panel). In the case of Pfs48/45^10C^, ELISA-positive sera from the rabbit described in Table 2 reacted with numerous proteins under both reducing and non-reducing conditions, however, reactivity with a band of the expected mass of Pfs48/45 was observed only under non-reducing conditions (Fig. 5B: highlighted with an asterisk). Lastly, sera from animals injected with Grl1p did not detect either Pfs25 in gamete extracts (Fig. 5A) or Pfs48/45 in gametocyte extracts (Fig. 5B).

### 3.6 Sera from immunized rabbits block transmission

As an initial screen to identify immunization conditions that would produce transmission-blocking antibodies, standard membrane feeding assays (SMFA) were carried out using total IgG purified from pre-immune and day 70 sera from individual Pfs25, Pfs25:Grl1p and Grl1p immunized animals that exhibited the highest end-point titers as shown in Table 2. For Pfs48/45^10C^:Grl1p immunized animals, IgG was purified from Day 70 sera from the single Montanide group animal that showed a specific anti-Pfs48/45 immune response as described in Table 2. For each sample in an SMFA experiment, mosquitoes were fed antibodies from a single animal (which had exhibited the highest titer in each group), and 20 mosquitoes were dissected and oocysts counted (Table 3). Antibodies from animals injected with Pfs25 + Alum conferred 98.0% transmission-blocking activity that was similar to the 98.6% blocking effect of the positive control 4B7 mAb. Antibodies from animals injected with either Pfs25:Grl1p + Alum or Pfs25:Grl1p + Montanide displayed significant (*p*-value <0.005) transmission blocking activity at 82.9% and 67.8% respectively. Antibodies from the rabbit injected with Pfs48/45^10C^:Grl1p + Montanide showed modest but non-significant (*p*-value >0.005) 39.4% transmission blocking. In contrast, control antibodies from a rabbit immunized with Grl1p did not block oocyst formation.

### 3.7 Manipulation of culture conditions further improves solubility of Pfs25:Grl1p

**Fig. 6:**
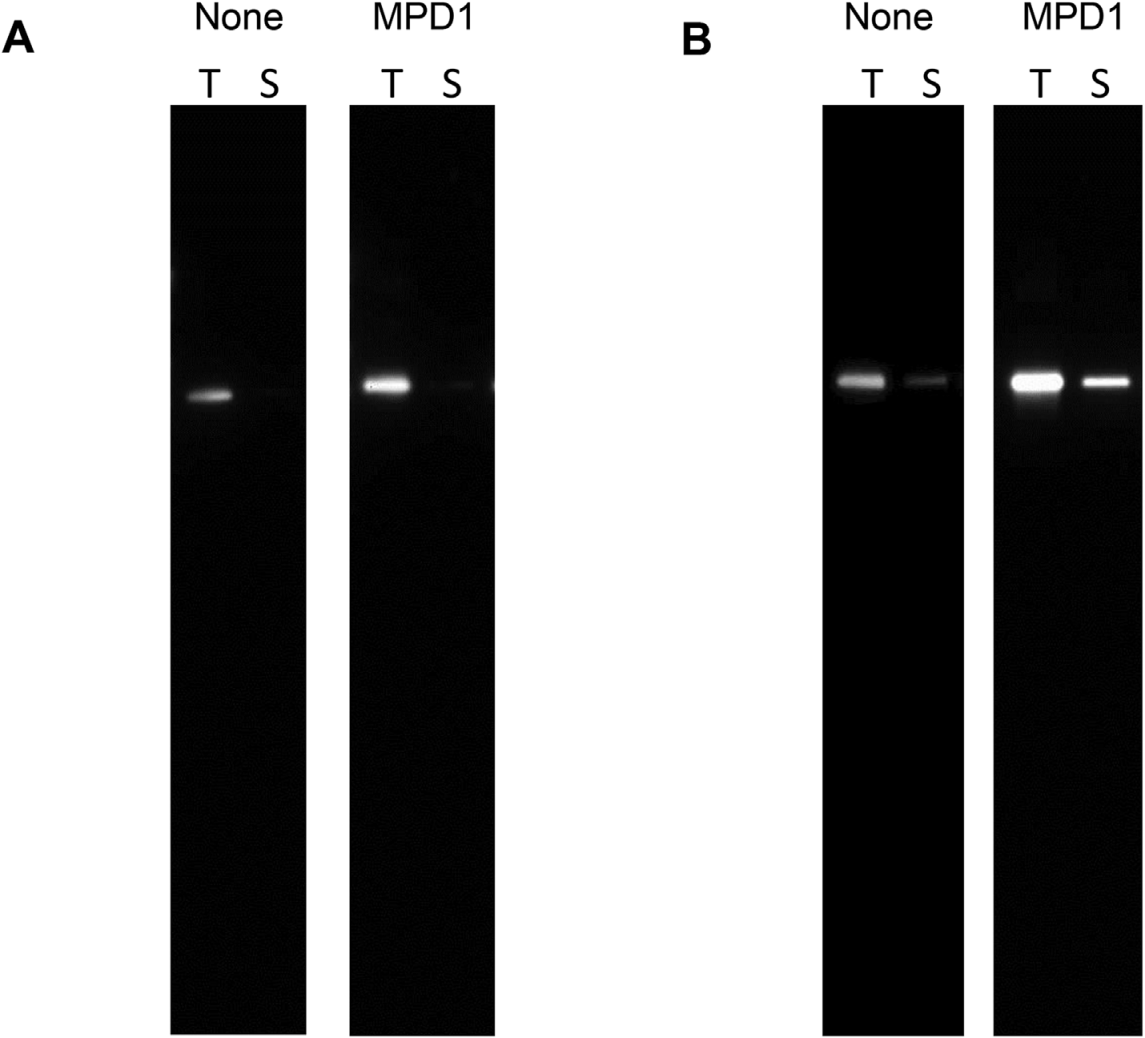
**Solubility of Pfs25:Grl1p in the absence and presence of MPD1**. Pfs25:Grl1p was expressed in *E. coli* in the absence or presence of MPD1. Equal volumes of total cell pellet (T) and soluble fraction (S) were analyzed by Western blot using an antibody against the C-terminal His tag. A. Expression at 30 °C. B. Expression at 16 °C.

As described, fusion of Pfs25 and Pfs48/45^*10C*^ to Grl1p greatly improved their solubility under non-optimized culture conditions. To see if we could further improve the yield of soluble Pfs25:Grl1p as a test case, we co-expressed Pfs25:Grl1p with individual chaperone proteins (BiP, DegP1(S210A), surA, fkpA, Tig-fkpA, MPD1, MPD2, or DsbC, see Materials and Methods). Co-expression at 30 °C or 16 °C resulted in a boost in total protein production, percent soluble protein, and/or yield of soluble protein in many cases (Table 4). The best results were observed with co-expression of the *S. cerevisiae* protein disulfide-isomerase MPD1 at 16 °C, which increased the yield of soluble protein by over 80-fold compared with no chaperone at 30 °C (50.1 mg versus 0.6 mg per liter) and by nearly 4-fold compared with no chaperone at 16 °C (50.1 mg versus 13.5 mg per liter). Fig. 6 shows a Western blot of the yield of soluble Pfs25:Grl1p in the absence and presence of MPD1 at 30 °C and 16 °C, illustrating the increase in total protein production and solubility of Pfs25:Grl1 when expressed at 16 °C and in the presence of MPD1.

## 4 Discussion

Although elusive, the need for effective, low-cost vaccines for malaria continues to exist. *E. coli* is a well-established platform for the production of human biologics and can potentially produce large amounts of proteins at extremely low cost. To date transmission blocking vaccine candidates such as Pfs25 and Pfs48/45 have been largely insoluble when expressed in *E. coli*. Here we demonstrate that Grl1p, a self-assembling protein from *Tetrahymena*, can act as a fusion tag to dramatically increase the solubility of both proteins. Standard manipulations of *E. coli* growth and induction conditions can often further improve the yield of correctly folded antigen. Pfs25 was more soluble and correctly folded when expressed in *P. pastoris* strains over-expressing protein disulfide isomerase [21]. In the case of Pfs25:Grl1p the yield of soluble protein increased by as much as 180-fold compared to Pfs25 alone, and co-expression of a chaperone increased this by at least 80-fold (with MPD1, see Table 4 and Fig. 6). The mechanism for the Grl1p-dependent solubilization enhancement is not known. The ability of fusion tags to confer solubility on their partner proteins may be due to the formation of micelle-like structures that sequester and protect unfolded or misfolded proteins, their ability to attract chaperones, their intrinsic chaperone-like activity, and/or their net charges [46]. The net negative charge of the Grl1p moiety (pI = 4.28) could reduce aggregation through electrostatic repulsion, as has been suggested for other acidic fusion partners that confer solubility [46,47]. Structures of the *E. coli* chaperone GroEL and the solubility-enhancing maltose-binding protein have identified hydrophobic clefts on their surface, which have been proposed to bind incompletely folded proteins [48]. It is unclear if Grl1p could be acting through this mechanism, as no structure currently exists. Grl1p is unique among well-known fusion tags such as maltose-binding protein and glutathione-S-transferase in containing predicted coiled-coil domains. Fusion to full-length Grl1p was necessary for maximum enhancement of solubility; fusions to truncated versions of Grl1p containing fewer predicted coiled-coil domains were much less soluble (A. Agrawal, unpublished results).

From a manufacturing standpoint, production of soluble antigens without the need for protein refolding represents a significant advantage. However solubility alone does not ensure that target antigens are correctly folded and immunogenic. Pfs25 and Pfs48/45 are complex proteins containing multiple disulfide bonds that form conformation-dependent transmission blocking epitopes required for vaccine efficacy. In Western blots with mAbs that recognize disulfide-restricted transmission blocking epitopes on the native proteins, substantial proportions of *E. coli* expressed Pfs25:Grl1p (∼40% with mAb 4B7) and Pfs48/45^10C^:Grlp (∼60% and ∼30% with mAbs 85RF45.1 and 85RF45.2b, respectively) were detected under non-reducing conditions. Furthermore, Pfs25:Grl1p was recognized by conformation-dependent antibodies in ELISA-based assays, confirming not only the presence of these epitopes but their accessibility in the context of the Grl1p fusion and G-SOME particles. Finally, it is worth noting that while a yeast-expressed Pfs25 antigen failed to react with the transmission blocking mAb, 4B7, under non-reducing conditions, it was nevertheless capable of eliciting a transmission blocking immune response in animals [20].

Recombinant Pfs25:Grl1p and Pfs48/45^10C^:Grl1p spontaneously formed particles in *E. coli*. In the case of Pfs25:Grl1p two particle size distributions of ∼38 and 106 nm, were detected by DLS. In *Tetrahymena,* granule lattice proteins, including Grl1p, are released from cells to form a proteinaceous gel that is highly resistant to elevated temperature [49]. We have found that continuous incubation of Pfs25:Grl1p at 40 °C results in progressive degradation of protein (∼75% loss over the course of a week, A. Agrawal, unpublished results), leaving a single particle population with a peak diameter of 38 nm. Interestingly this coincided with an increase in the percent of protein recognized by mAb 4B7 under non-reducing conditions from ∼25% at day 0 to close to 62% on day 7 (A. Agrawal, unpublished results). Thus, in addition to improved solubility, fusion to Grl1p may stabilize correctly folded versions of some proteins. While most vaccines are licensed for storage at 2–8 °C regardless of their thermostability [50], increased stability can reduce costs and obviate the need for a “controlled temperature chain”. MenAfriVac, for example, a monovalent conjugate vaccine against meningococcal serogroup A licensed for use in mass vaccination campaigns, is effective after storage at up to 40 °C for up to four days [51] leading to an estimated 50% reduction in storage and transport costs [52].

Both Pfs25 and Pfs25:Grl1p generated similar levels of anti-Pfs25-antibody production, signifying no obvious Grl1p-dependent effects on the rabbit immune response to the Pfs25 moiety. Similarly, the quality of the antibody response appeared to be similar in both cases: IgGs from animals vaccinated with either Pfs25 alone or Pfs25:Grl1p recognized native Pfs25 antigen in gamete extracts only under non-reducing conditions (similar to known transmission blocking antibodies e.g. 4B7), and each significantly blocked oocyst development (>80%, *p*-value <0.005) in mosquitoes. In animals injected with Pfs25:Grl1p similar antibody titers were seen against the Pfs25 target and the fusion tag indicating that the immune response was equally directed against each of the fusion partners. The highest titer responses were limited to immunogens formulated with either Alum or Montanide and differences in the responses to Pfs25 alone versus Pfs25:Grl1p were negligible suggesting that the particulate nature of the fusion protein had no adjuvant affect. In the case of Pfs48/45^10C^, a small yet specific anti-Pfs48/45 immune response was noted only in one animal (injected with Pfs48/45^10C^:Grl1p + Montanide). Antibodies from that animal showed modest transmission-blocking activity (39.4%), consistent with the low level of specific reactivity on a Western blot with native Pfs48/45 in a gametocyte extract. Conversely, high anti-Grl1p antibody titers were observed in most Pfs48/45^10C^:Grl1p vaccinated animals indicating that, for this protein, a majority of the immune response was directed against the Grl1p moiety. This is in contrast to other nanoparticle-based vaccines involving protein fusions, where the antigen is displayed in a repetitive fashion on the surface and the fusion partner is somewhat buried [53,54]. In the case of Pfs25:Grl1p and Pfs48/45^10C^:Grl1p, the similar or greater magnitude of the antibody response against the Grl1p component compared with the antigen component suggests that the proteins do not form such a structure. Co-crystal structures of transmission-blocking antibodies (generated against a plant-produced Pfs25 VLP in human immunoglobulin loci transgenic mice) with Pfs25 produced in human cells showed that the antibodies recognized two distinct immunogenic sites, one of which broadly overlapped with the 4B7 antibody epitope [14]. These sites did not overlap with the binding site of the transmission-blocking antibody co-crystallized with Pvs25 [15]. The epitopes recognized by our polyclonal antibodies are unknown, although they include at least some transmission-blocking epitopes as shown by the results of the membrane-feeding assay. Nevertheless, it is difficult to predict the nature or structure of the fusion protein based on the two we have investigated. Both proteins are similar in being GPI-anchored surface proteins. The size of the protein or number of cysteines does not appear to predict correct folding or antigenicity, as Pfs25 has 22 cysteines over 172 amino acids (11 disulfide bonds) [13,14] while Pfs48/45^10C^ has 10 cysteines over 268 amino acids. The elucidation of a high-resolution structure of an antigen-carrying G-SOME particle may enable structure-guided vaccine design to i) increase antigen accessibility and uniformity on the particle surface with a potential concomitant increase in potency and/or ii) a decrease in the accessibility of the Grl fusion partner to mitigate off-target immune responses.

Antigen-fusion proteins are not a new concept in recombinant vaccine design. Pfs25 fused to *Salmonella* flagellin was soluble in *E. coli*, though antibodies generated against the protein were not assessed for transmission-blocking activity [55]. The current leading malaria transmission blocking vaccine candidates are fusion proteins designed to boost antigen immunogenicity. *P. pastoris* Pfs25 conjugated to a detoxified form of *Pseudomonas aeruginosa* exoprotein A produced in *E. coli* has been tested in a Phase I clinical trial with good immunogenicity and transmission blocking [56]. *P. pastoris*-produced Pfs25 chemically conjugated to the outer-membrane protein complex of *Neisseria meningitidis* serogroup B was more immunogenic than Pfs25 alone [26]. Additionally, a vaccine consisting of virus-like particles (VLPs) of Pfs25 fused to the coat protein of alfalfa mosaic virus and produced in tobacco VLP plants has exhibited potent immunogenicity and transmission blocking *in vivo* [27]. Pfs25 fused to the nanoparticle-forming protein IMX313 and produced from viral vectors or in *P. pastoris* showed better antibody responses in mice and better transmission-blocking activity compared with monomeric Pfs25 [28]. Development of a malaria transmission blocking vaccine will likely favor the use of simple technologies to produce vaccines at a low cost of goods sold [57]. Our approach takes advantage of a simple expression platform where fusion to *Tetrahymena* Grl1p generates large amounts of soluble protein in *E. coli* from largely insoluble proteins without the need for protein refolding. With the potential added benefit of heat-stability, fusion to Grl1 can act as a solubility enhancer for proteins with limited solubility while retaining correct folding, which may be useful for applications such as the production of vaccines and other biologics.
